# Dietary changes needed to improve diet sustainability: are they similar across Europe?

**DOI:** 10.1038/s41430-017-0080-z

**Published:** 2018-02-05

**Authors:** Florent Vieux, Marlene Perignon, Rozenn Gazan, Nicole Darmon

**Affiliations:** 1 MS-Nutrition, Marseille, France; 2 UMR MOISA, Inra 1110, CIRAD, SupAgro, CIHEAM-IAMM, Université de Montpellier, MOISA (Markets, Organizations, Institutions and Strategies of Actors), 34060 Montpellier 2, France

**Keywords:** Nutrition, Epidemiology

## Abstract

**Background/objectives:**

It is not known whether dietary changes able to simultaneously achieve nutritional adequacy and reduce diet-related greenhouse gas emissions (GHGE) are similar across Europe when cultural and gender specificities are taken into account.

**Subjects/methods:**

Starting from each mean observed diet in five European countries (France, UK, Italy, Finland, and Sweden) and for each gender, nutritionally adequate diets departing the least from observed diet were designed with linear programming by applying stepwise 10% GHGE reductions. Other models directly minimized GHGE.

**Results:**

For most countries and whatever the gender, achieving nutritional adequacy implied between-food-group subtitutions (i.e., replacing items from the sugar/fat/alcohol food-group with items from the fruit and vegetables and starchy food-groups), but increased GHGE. Once nutritional adequacy was met, to decrease GHGE, the optimization process further induced within-food-groups substitutions that were reinforced by stepwise GHGE reductions. Diet modeling results showed the need for changes in consumption of animal-based products but those changes differed according to country and gender, particularly for fish, poultry, and non-liquid milk dairy. Depending on country and gender, maximal GHGE reductions achievable ranged from 62% to 78% but they induced large departures from observed diets (at least 2.8 kg/day of total absolute weight change) by modifying the quantity of at least 99% of food items.

**Conclusions:**

Setting nutritional goals with no consideration for the environment may increase GHGE. However, diet sustainability can be improved by substituting food items from the sugar/fat/alcohol food group with fruit, vegetables, and starches, and country-specific changes in consumption of animal-based products. Standardized surveys and individual diet modeling are promising tools for further exploring ways to achieve sustainable diets in Europe.

## Introduction

The Food and Agriculture Organization (FAO) defines sustainable diets as “protective and respectful of biodiversity and ecosystems, culturally acceptable, accessible, economically fair and affordable, nutritionally adequate, safe and healthy, while optimizing natural and human resources” [1]. In the European Union, food consumption was estimated to be responsible for 20–30% of the total environmental impacts of total household consumption [2].

A number of studies have assessed the environmental impact of current diets or dietary shifts [3, 4], most using greenhouse gas emissions (GHGE) as an environmental indicator. These studies nearly always find that meat and dairy are among the largest contributors to GHGE, whereas high consumption of vegetables, fruits, and legumes/pulses/nuts is associated with the lowest GHGE [5–7]. For example, Temme et al. [6] indicated that in the Netherlands, meat and cheese contributed to about 40% of the GHGE daily diets when potatoes, vegetables, and fruits contributed to 9%. These results are consistent with the idea that reducing meat consumption would benefit both health and the environment, as worldwide growth in meat output exacerbates climate change and increases the risk of certain non-communicable diseases [8]. Several studies have investigated the potential of alternative diets to reduce the environmental impact of food consumption by comparing the environmental impact of observed mean diets against hypothetical dietary scenarios (e.g., vegetarian, vegan, or flexitarian) [9–12] or dietary pattern scenarios considered more healthful and regionally acceptable, such as the Mediterranean and New Nordic diets [13–16]. In most of these studies, scenarios of diets with less animal products have a lower environmental impact than observed diets [9–13, 15, 16]. However, hypothetical dietary scenarios are based on a priori decisions and are not representative of actual food consumption in terms of food choices and energy content, and thus ignore the cultural acceptability factor. Furthermore, their assessment of dietary quality do not consider the full set of nutritional requirements.

The concept of diet sustainability implies to integrate simultaneously the environmental impact, nutritional adequacy, economic affordability, and cultural acceptability of diets. This kind of integrative approach can be conducted using linear programming, which is a unique tool for producing diet models that consider the multifactorial aspect of diet sustainability issues by testing the feasibility of complex problems involving multiple variables and constraints and finding their optimal solution [17–19]. A French study based on linear programming found that moderate GHGE reductions (≤30%) were compatible with nutritional adequacy and affordability without important food-group shifts from observed diets [20]. Research in the UK showed that a healthy diet with reduced GHGE (−27%) is possible but would entail substantive dietary changes for a majority of individuals [21]. In both studies, diets designed with an imposed GHGE reduction involved a lower content of foods of animal origin, with the exception of fish [20]. However, all the above studies were country-specific and used heterogeneous modeling methods or GHGE assessments, making appropriate comparison between countries impossible. Applying a harmonized modeling approach to several European country-specific food data consumption would contribute to derive a European policy for improving diet sustainability.

The main objective of the present study was to explore and compare the dietary changes needed to achieve a nutritionally adequate diet with lower GHGE across five European countries.

## Materials and Methods

### Study population

Dietary intake data were derived from national food consumption surveys to get a representative sample for each of the five countries studied, i.e., the national FINDIET 2012 Survey in Finland based on 48 h recall with two replicates (*n* = 1708) [22]; the Riksmaten 2010 study in Sweden based on 4-day dietary records (*n* = 1 797) [23]; the INRAN-SCAI-2005 study in Italy based on 3-day dietary records (*n* = 3323) [24]; the NDNS rolling program for 2008–2012 in the UK based on 4-day dietary records (*n* = 4156) [25]; the INCA2 study 2006–2007 in France based on 7-day dietary records (*n* = 4079) [26]. After removing individuals aged <18 years and >64 years and consumers of dietary supplements, the final samples were 569 men and 679 women in Finland, 930 men and 1323 women in France, 967 men and 1105 women in Italy, 588 men and 764 women in Sweden; and 627 men and 751 women in the UK. FINDIET study was approved by the institutional review board of the Helsinki University Hospital. The Riksmaten study was approved by the Regional Ethical Review Board of Uppsala. The INRAN-SCAI 2005 survey was exclusively observational and non-invasive. INRAN is part of the National Statistical System and guarantees individual data protection. NDNS study was approved by the Oxfordshire A Research Ethics Committee. INCA2 was approved by the French authority of data protection and the French national council for statistical information. Informed consent (oral or written) was obtained from all participants and formally recorded.

### Food databases

A common food classification derived from the FoodEx classification system [27] was used for all five countries. After removing “Food for infants and small children” and “Products for special nutritional use”, the FoodEx classification was adjusted to better take account of both environmental and nutritional diet dimensions, resulting in a final nomenclature of 151 food items (Supplemental Table 1) grouped into 27 L1 FoodEx groups (L1 groups) and 10 main food groups to chart the results (Supplemental Table 1). Nutrient composition of the 151 food items was estimated for each country and gender by weighting the nutritional composition of the different foods composing the food item by their respective quantities consumed.

A GHGE estimate, expressed in grams of CO_2_ equivalents (g CO_2_eq), was assigned to each of the 151 food items as described by Hartikainen & Pulkkinen [28].

### Diet modeling with linear programming

Borrowing from Perignon et al. [20], linear programming models were developed to design nutritionally adequate diets with reduced GHGE while remaining as close as possible to the observed mean diets of the national populations. Diets were modeled for each country, at increasingly stringent levels of GHGE reduction, and for men and women separately. All linear programming models were run using the SAS v9.4 statistical software package. Computer code can be accessed on demand to corresponding author.

#### Nutritional constraints

Nutritional adequacy was defined by the fulfillment of a set of 32 nutrient recommendations, which were derived from the dietary reference values published by the European Food Safety Agency (EFSA) [29] and completed by maximum intake of sodium and saturated fatty acids derived from the Nordic nutrient recommendations [30]. Maximum amount of free sugars was derived from the World Health Organization recommendations [31]. Table 1 charts the entire set of nutrient recommendations applied to every country-specific model.

**Table 1:** Nutrient constraints applied to the linear programming models, derived from the European Food Safety Agency (EFSA) dietary reference values, unless specified

	Nutrient recommendations	
	Women	Men
Proteins (g/body weight^a^)	>0.83	>0.83
Carbohydrates (% E)	(45–60)	(45–60)
Fats (% E)	(20–35)	(20–35)
SFA^b^ (% E)	<10	<10
Free sugars^c^ (% E)	<10	<10
Sodium^b^ (mg/d)	(575–2400)	(575–2400)
Fiber (g/day)	>25	>25
Calcium (mg/day)	(950–2500)	(950–2500)
Magnesium (mg/day)	>300	>350
Phosphorus (mg/day)	>550	>550
Iron (mg/day)	>16	>11
Potassium (mg/day)	>3100	>3100
Zinc (mg/day)	(10.1–25)	(12.85–25)
Vitamin A (μg RE/day)	(650–3000)	(750–3000)
Thiamin (mg/day)	>0.9	>1.1
Riboflavin (mg/day)	>1.3	>1.6
Vitamin B6 (μg/day)	(1.1–25)	(1.5–25)
Vitamin B12 (μg/day)	>4	>4
Folic acid (μg/day)	(330–1000)	(330–1000)
Vitamin C (mg/day)	>95	>110
Vitamin D (μg/day)	(5–100)	(5–100)
Vitamin E (mg/day)	(11–300)	(13–300)

*% E* % of total energy intake, *SFA* saturated fatty acids

^a^Mean body weight was estimated for men and women separately based on national dietary survey data

^b^Nordic nutrient recommendation

^c^WHO 2015

#### Acceptability constraints

To avoid unrealistic dietary changes, total food weight was constrained to within ±20% of total observed diet weight, and diet weight per food item and L1 food group were limited to between the 10th percentile observed in the whole population (including non-consumers) and the 90th percentile among consumers. Ratio of solid-to-liquid food weights (including ready-to-eat soups, liquid milk), maximal amounts of alcoholic beverages, fish oil and offal (of meat or fish), and total energy intakes were set to the observed values.

#### Environmental constraints

For each country and gender, diets were modeled under the nutritional and acceptability constraints described above, and a stepwise increase in GHGE reduction was imposed as follows: (i) a nutritionally adequate diet with minimized departure from national mean observed diet without constraint on GHGE (NUTR) to identify the dietary changes induced by the achievement of nutritional recommendations only, and to test whether improving nutrition will likely improve the environmental dimension, (ii) several nutritionally adequate diets with minimized departures from the national mean observed diet with a GHGE constraint imposing a stepwise (10% steps) GHGE reduction (NUTR-GHGE), starting from the GHGE value of the mean observed diet up to the maximal 10% step achievable, and (iii) a nutritionally adequate diet with minimized GHGE (NUTR-GHGE-MINI) to test what would be the maximal achievable GHGE reduction under the nutritional constraints.

#### Objective function

For all models run except those minimizing GHGE, the objective function minimized the total departure between the observed diet and its corresponding modeled diet. Total departure was minimized by minimizing the objective function *f*, expressed as the sum of absolute values of the relative weight change for each food item:

$$\mathrm{Minimize}\,f{\mathrm{ = }}\mathop {\sum }\limits_{i = 1}^n \mathrm{ABS}\left( {\frac{{Q_i^{\mathrm{opt}} - Q_i^{\mathrm{obs}}}}{{Q_i^{\mathrm{obs}}}}} \right),$$

where *i* is a food item, *n* is number of available food items in the country and gender population modeled, *Q*^opt^ is optimized quantity, and *Q*^obs^ is mean observed quantity.

### Data analysis

For each model, we assessed departure from observed diet in terms of mean absolute quantity variation of food items (%) as well as proportion of food items for which quantity was modified. We then focused specifically on the dietary changes needed to achieve a 30% GHGE reduction, as it was previously suggested (based on French data) that a higher increase in GHGE reduction would impair the acceptability of changes [20].

## Results

### Total GHGE and energy content of observed, NUTR, and NUTR-GHGE-MINI modeled diets

In the observed diets, energy content ranged from 1591 kcal/day (resp. 2109 kcal/day) to 1888 kcal (resp. 2360 kcal) in women (resp. in men, Table 2). GHGE ranged from 3403 g CO_2_eq/day (resp. 4636) in the UK to 4321 in France (resp. 5793) in women (resp. men). For women, fulfilling the whole set of nutrient recommendations induced an increase in GHGE in the modeled diets, except in the UK. For men, the same increase in GHGE was seen for all countries except Italy and Finland. Depending on the gender and country, the minimum achievable GHGE ranged from 954 g CO_2_eq/day to 2224 g CO_2_eq/day, hence, a maximal reduction ranging from 62 to 78%. However, such reductions in GHGE were associated with substantial departures from observed intakes, illustrated by a quantity variation per food item that ranged from 837–4703%.

**Table 2:** Total GHGE, diet weight, energy content in observed diets, and variations in quantities and GHGE induced by NUTR and NUTR- GHGE-MINI models

	France	UK	Italy	Finland	Sweden
*Observed diets*					
GHGE, in g CO_2_eq/day					
Women	4321	3403	4152	3890	3864
Men	5793	4636	5145	5191	5254
Diet weight, in g/day					
Women	2465	2444	2062	3067	2563
Men	2720	2935	2244	3345	2792
Energy content, in kcal/day					
Women	1783	1591	1888	1720	1778
Men	2360	2109	2319	2223	2260
*NUTR model*					
GHGE, in g CO_2_eq/day (and % variation from observed diet)					
Women	5004 (+16%)	3138 (−8%)	5812 (+40%)	5508 (+42%)	5645 (+46%)
Men	7464 (+29%)	6086 (+31%)	4976 (−3%)	4253 (−18%)	6022 (+15%)
Mean absolute quantity variation of food items (%)					
Women	43.23	148.27	41.95	46.41	70.69
Men	30.64	37.86	16.03	31.99	23.54
*NUTR-GHGE-MINI model*					
GHGE, in g CO_2_eq/day (and % variation from observed diet)					
Women	1590 (−63%)	1213 (−64%)	1256 (−69%)	954 (−75%)	1362 (−65%)
Men	2224 (−62%)	1256 (−73%)	1623 (−68%)	1120 (−78%)	1328 (−75%)
Mean absolute quantity variation of food items (%)					
Women	1747	2445	3629	2657	837
Men	988	2201	4703	2255	921

### Changes needed at food-group level

**Fig. 1:**
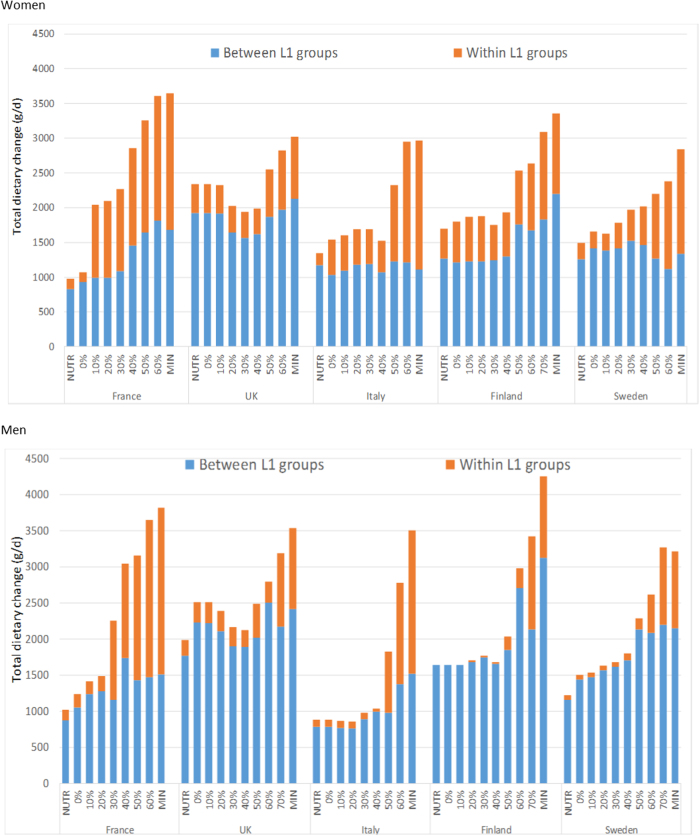
Total absolute weight changes (in g/day) induced by NUTR, every NUTR-GHGE (by steps of 10% GHGE reduction) and NUTR-GHGE-MINI models, separated into within- and between-L1 food-group substitutions

Total diet weight and quantities of main food groups and L1 food groups in OBS, NUTR and NUTR-GHGE-30% diets are presented in Supplemental Table 2. For most countries and whatever the gender, achieving nutritional adequacy (NUTR model) implied between food-group substitutions (i.e., replacing items from the sugar/fat/alcohol food group with items from the fruit and vegetables and starchy food groups), but increased GHGE. This GHGE increase was associated with an increase in total diet weight. At least 1 kg/day (except in Italy for men and France for women) of total absolute weight change was needed to reach a nutritionally adequate diet (Fig. 1). Women needed larger food changes than men to meet their nutrient recommendations only, except in France and Finland where total absolute weight change changes (in grams) were equivalent between genders. In all countries and for both genders, the majority of dietary changes needed to reach a nutritionally adequate diet were substitutions between-food groups. This is particularly true among men, for whom around 90% of the dietary changes needed to reach an adequate diet were between-food groups (compared to at least 70% for women). Then, for each NUTR-GHGE model and for each country and gender, the share of within-food-group substitutions in absolute weight change increased with decreasing GHGE, but not necessarily in a linear way.

### Changes needed at food-item level

**Fig. 2:**
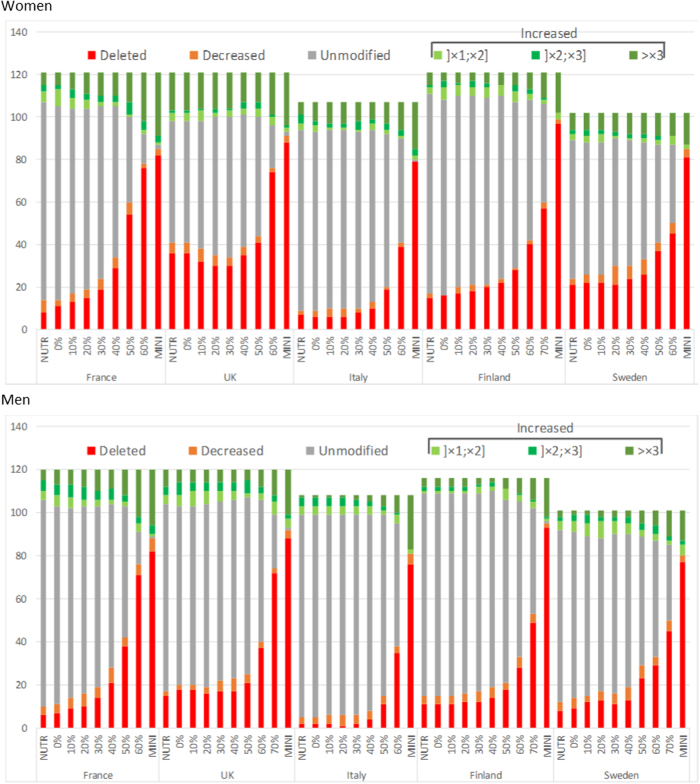
Number of food-items whose quantity was decreased, increased, unmodified, or deleted in NUTR, each NUTR-GHGE (by steps of 10% GHGE reduction) and NUTR-GHGE-MINI models, in comparison to observed diets

For both genders, the majority of food-item quantities did not need to change to meet nutrient recommendations when no constraint was applied on GHGE (NUTR model), except for women in the UK for whom quantities needed to change for 53% of food items (Fig. 2). Then, applying a constraint to stepwisely decrease GHGE increased the number of foods whose quantity needed to change. Different food change trends emerged as the GHGE reduction constraint progressively increased. Whatever the country or gender, minimizing GHGE induced a modification in quantity of at least 99% of food items.

### Food-group contributions to energy and GHGE with the NUTR-GHGE-30% model

**Fig. 3:**
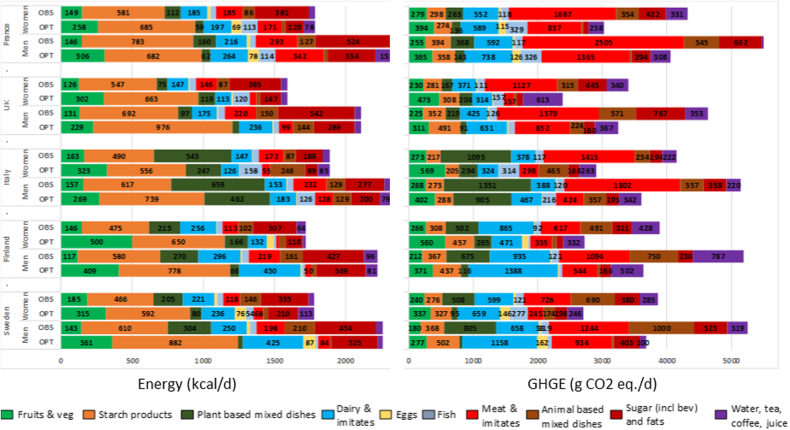
Contributions of the main food groups to energy content (kcal/day) and GHGE (g CO_2_eq/day) in observed (OBS) and nutritionally adequate diets modeled with a 30% reduction in GHGE (NUTR-GHGE-30%). For clarity purposes, values <50 kcal and <100 g CO_2_ were removed

Whatever the country or gender, compared to the observed diet, achieving nutritional adequacy with a 30% reduction of GHGE (NUTR-GHGE-30%) increased energy and GHGE from fruit and vegetables, and decreased energy and GHGE from the sugar/fat/alcohol food group (Fig. 3). The contribution of fish to energy and GHGE increased in modeled diets for all countries except Finland (both genders), Sweden and the UK (men only). French men were the only population for which contribution of meat to energy increased, but within-meat-group substitutions led to a decrease in contribution of meat to GHGE in this population: while beef, lamb, preserved meat, and sausage were suppressed, poultry and pork quantities increased (data not shown). The main contributor to GHGE was meat in all observed diets, except the diets of Finnish women for whom the main GHGE contributor was dairy. Meat remained the main contributor in modeled diets for men and women in France and for men in UK.

## Discussion

This modeling study identified the dietary changes required for each gender to reach a nutritionally adequate diet while reducing diet-related GHGE in five European countries (Finland, France, Italy, Sweden, the UK). In agreement with previously published studies, it showed that nutritional adequacy was not necessarily associated with reduced GHGE [20, 32], which could be related to the higher diet weight of nutritionally adequate low energy-dense modeled diets [3]. The study also showed that a maximal GHGE decrease of 62–78% was theoretically achievable while still ensuring nutritionally adequacy, but at a strong risk of compromising the cultural acceptability of the diets [20]. Moreover, this study highlighted the similarities and country and gender specificities of the dietary changes needed across Europe to move toward more sustainable diets. Among other similarities observed across countries, between-food-group substitutions were needed to reach a nutritionally adequate diet, after which within-food-group substitutions were needed to reduce GHGE. We also found that an increased contribution of energy from fruits, vegetables and starchy foods and a decreased contribution of energy from sugar and fats were also needed for all countries and genders, which is consistent with several previous modeling studies [20, 33–35] and with general dietary advice [36]. More country and/or gender specificities were identified for animal-based products. Not only energy coming from fish varied differently across countries (increasing in France and Italy and decreasing in Finland for both genders) but also between men and women within countries (increasing in women and decreasing in men in both the UK and Sweden). Previous population-based linear programming studies have found that fish quantity needed to be increased to reduce GHGE of diet in France [20] and in UK women [33], which is consistent with the present results (supplemental Table 2). Although energy coming from dairy increased for both genders in Sweden and France, it increased in men but decreased in women in Finland, Italy, and UK. However, a common increase in liquid milk was found whatever the gender or country (except Italian men for whom it remained unchanged), which is in line with what has been previously been found in France [20] and the UK [34]. The increase in energy coming from meat in French men’s diet was unexpected since meat, particularly red meat, is known to be the food group with the greatest environmental impact [5–7]. However, this result warrants careful interpretation, as only poultry and pork, i.e., the least impacting food items (in terms of GHGE) within the non-processed meat group, were found to increase in the diet modeled for French men.

The dietary changes induced by diet modeling are driven by three input characteristics: specific dietary habits (which differ between countries and gender and directly correlate with both nutritional quality and GHGE), nutritional needs (which differ between men and women) and nutritional quality of the offer (which differs mainly between countries). UK women are the target population for which modeled diet was most affected by specific dietary habits. Their food habits were associated with the lowest GHGE compared with other countries and gender, but also with the most inadequate intakes of magnesium, vitamin E, vitamin C, folates, zinc, iron, calcium, potassium, fibers, and free sugars (data not shown). Consequently, this target group needed to make the biggest dietary changes to achieve a nutritionally adequate diet, whereas at the same time being the most environmentally constrained given that GHGE reduction was expressed as a percentage of observed level.

Differences between dietary changes required for men and women can be explained by differences in nutritional needs. As an example, while recommended iron intake was achieved by men in the mean observed diet in each country, it was never achieved among women. Consequently, in NUTR-model diets, the quantity of iron-rich foods, such as breakfast cereals, was at least tripled in all diets modeled for women but remained stable for men in Italy, Finland, and Sweden.

The last factor that may explain between-population differences in dietary changes is nutritional content of the offer. To illustrate, nutrient composition of prepared salads was not the same between countries and was particularly low in nutrients to encourage in Sweden. Sweden is consequently the only country for which quantity of prepared salad decreased in NUTR-GHGE-30% diets.

This study carries certain limitations in terms of comparability of the results across countries. First, the methods used for dietary data collection were different, which makes direct country-to-country comparison difficult. Only detailed food consumption data derived from a harmonized methodology across Europe would allow comparisons between countries as planned by European and international organizations [37]. Second, the population-based analysis hindered any statistical evaluation of dietary changes induced by the modeling exercise. An alternative and more robust approach would be to conduct individual-based linear programming by modeling an optimized diet for each individual, which can also integrate individual food preferences, as already applied in France [38] and the UK [21].

Moreover, the fact that the environmental dimension of sustainable diet was represented solely by the GHGE metrics can be seen as a limitation. Assessment of the environmental dimension should be enriched by other metrics, such as eutrophication, water footprint, land use or biodiversity indicators, which have all been shown to not necessarily correlate positively with GHGE [39, 40]. As an example, GHGE indicator does not reflect sustainability concerns induced by an increase in the consumption of fish (overfishing, loss of biodiversity…). Comprehensive databases that integrate a massive number of metrics representing sustainability as a whole would therefore be desirable and would perfectly fit with diet modeling approaches [41]. Finally, even if the geographic position of selected countries is heterogeneous, the application of this methodology to other European countries would allow to increase knowledge on dietary habits able to improve sustainability. This assumes an increase in accessibility to food related dataset.

## Conclusion

This population-based modeling study showed that setting nutritional goals with no consideration for the environment may increase GHGE, and that there are country and gender specificities when studying the smallest dietary changes needed to reach a nutritionally adequate diet with lower GHGE. Although an increase in plant-based products was needed in every countries, the shifts in animal-based products were not homogeneous across countries or gender. These results now need to be confirmed and extended, especially by using individual-based modeling and homogeneously designed dietary surveys. Such study would allow the EFSA to provide strong recommendations to member states authorities when developing their own guidelines for sustainable food choices.

## Electronic supplementary material

Final nomenclature(DOCX 24 kb)

Contributions of L1 food groups to total diet weight (in grams/day) in observed (OBS), nutritionally adequate diets (NUTR) and nutritionally-adequate diets modeled with a 30% reduction in GHGE(DOCX 48 kb)
