# Supplementary material for: Dietary changes needed to improve diet sustainability: are they similar across Europe?
Source: Eur J Clin Nutr. 2018 Feb 5;72(7):951–60. doi: 10.1038/s41430-017-0080-z (PMC6035144; doi:10.1038/s41430-017-0080-z)
Supplement: Supplementary file 1 — Final nomenclature(DOCX 24 kb) [file 41430_2017_80_MOESM1_ESM.docx]

**Supplemental Table 1. Final nomenclature**

| **Main food groups**  **(N=10)** | **L1 food groups**  **(N=27)** | **Food-items**  **(N=151)** |
| --- | --- | --- |
| **Fruits &**  **vegetables** | Vegetables and  vegetable products | Vegetables and vegetable products (unspecified) |
|  |  | Root vegetables |
|  |  | Bulb vegetables |
|  |  | Fruiting vegetables |
|  |  | Brassica vegetables |
|  |  | Leaf vegetables |
|  |  | Legume vegetables |
|  |  | Stem vegetables (fresh) |
|  |  | Sugar plants |
|  |  | Sea weeds |
|  |  | Tea and herbs for infusions (solid) |
|  |  | Cocoa beans and cocoa products |
|  |  | Coffee beans and coffee products (solid) |
|  |  | Coffee imitates (solid) |
|  |  | Vegetable products |
|  |  | Fungi, cultivated |
|  |  | Fungi, wild, edible |
|  | Legumes, nuts and  oilseeds | Legumes, nuts and oilseeds (unspecified) |
|  |  | Legumes, beans, green, without pods |
|  |  | Legumes, beans, dried |
|  |  | Tree nuts |
|  |  | Oilseeds |
|  |  | Other seeds |
|  | Fruit and  fruit products | Fruit and fruit products (unspecified) |
|  |  | Citrus fruits |
|  |  | Pome fruits |
|  |  | Stone fruits |
|  |  | Berries and small fruits |
|  |  | Oil fruits |
|  |  | Miscellaneous fruits |
|  |  | Dried fruits |
|  |  | Jam, marmalade and other fruit spreads |
|  |  | Other fruit products (excluding beverages) |
| **Starch products** | Grains and  grain-based products | Grains and grain-based products (unspecified) |
|  |  | Grains as crops |
|  |  | Grains for human consumption |
|  |  | Grain milling products |
|  |  | Bread and rolls |
|  |  | Pasta (Raw) |
|  |  | Breakfast cereals |
|  |  | Fine bakery wares |
|  | Starchy roots  and tubers | Potatoes and potatoes products |
|  |  | Other starchy roots and tubers |
| **Plant-based**  **mixed dishes** | Vegetable composite  dishes | Composite food (unspecified) |
|  |  | Cereal-based dishes |
|  |  | Rice-based meals |
|  |  | Potato based dishes |
|  |  | Beans-based meals |
|  |  | Vegetable-based meals |
|  |  | Mushroom-based meals |
|  |  | Ready-to-eat soups |
|  |  | Prepared salads |
|  |  | Cheese-based meals |
| **Dairy &**  **imitates** | Dairy products | Dairy products (unspecified) |
|  |  | Liquid milk |
|  |  | Milk based beverages |
|  |  | Concentrated milk |
|  |  | Whey and whey products (excluding whey cheese) |
|  |  | Cream and cream products |
|  |  | Fermented milk products |
|  |  | Milk derivatives |
|  | Cheese | Cheese |
|  | Dairy imitates | Dairy product imitates |
| **Eggs** | Eggs and  egg products | Eggs and egg products (unspecified) |
|  |  | Eggs, fresh |
|  |  | Eggs, powder |
| **Fish** | Fish and  other seafood | Fish and other seafood (unspecified) |
|  |  | Fish products |
|  |  | Fish offal |
|  |  | Crustaceans |
|  |  | Water mollusks |
|  |  | Amphibians, reptiles, snails, insects |
|  |  | Tuna canned |
|  |  | Tuna not canned |
|  |  | Salmon |
|  |  | Cod |
|  |  | Other fatty fish |
|  |  | Other non-fatty fish |
| **Meat**  **& imitates** | Livestock  meat | Beef |
|  |  | Pork |
|  |  | Lamb |
|  |  | Livestock meat, other |
|  | Poultry | Poultry |
|  | Processed meat | Preserved meat |
|  |  | Sausages |
|  |  | Meat specialties |
|  |  | Pastes, pâtés and terrines |
|  | Meat imitates | Meat imitates |
|  | Other meat | Meat and meat products (unspecified) |
|  |  | Game mammals |
|  |  | Game birds |
|  |  | Mixed meat |
|  |  | Edible offal, farmed animals |
| **Animal-based mixed dishes** | Animal composite  dishes | Meat-based meals |
|  |  | Fish and seafood based meals |
|  |  | Egg-based meal (e.g., omelet) |
| **Sugar/fat/alcohol** | Sugar and  confectionary | Sugar and confectionary (unspecified) |
|  |  | Sugars |
|  |  | Sugar substitutes |
|  |  | Chocolate (Cocoa) products |
|  |  | Confectionery (non-chocolate) |
|  |  | Dessert sauces |
|  |  | Molasses and other syrups |
|  |  | Honey |
|  | Animal fats | Animal fat |
|  |  | Fish oil |
|  | Vegetable fats | Vegetable fat |
|  |  | Vegetable oil |
|  |  | Fats of mixed origin |
|  |  | Margarine and similar products |
|  | Alcoholic  beverages | Alcoholic beverages (unspecified) |
|  |  | Beer and beer-like beverage |
|  |  | Wine |
|  |  | Fortified and liqueur wines |
|  |  | Wine-like drinks (e.g. cider, perry) |
|  |  | Liqueur |
|  |  | Spirits |
|  |  | Alcoholic mixed drinks |
|  | Herbs, spices  and condiments | Herbs, spices and condiments (unspecified) |
|  |  | Herbs |
|  |  | Spices |
|  |  | Herb and spice mixtures |
|  |  | Seasoning or extracts |
|  |  | Condiment |
|  |  | Dressing |
|  |  | Chutney and pickles |
|  |  | Savory sauces |
|  |  | Flavorings or essences |
|  |  | Baking ingredients |
|  | Snacks, desserts,  and other foods | Snacks, desserts, and other foods (unspecified) |
|  |  | Snack food |
|  |  | Ices and desserts |
|  |  | Other foods |
|  | Soft drinks | Soft drinks |
|  | Alcoholic  beverages | Alcoholic beverages (unspecified) |
|  |  | Beer and beer-like beverage |
|  |  | Wine |
|  |  | Fortified and liqueur wines |
|  |  | Wine-like drinks (e.g. cider, perry) |
|  |  | Liqueur |
|  |  | Spirits |
|  |  | Alcoholic mixed drinks |
| **Water, tea, coffee, juice** | Fruit and  vegetable juices | Fruit and vegetable juices (unspecified |
|  |  | Fruit juice |
|  |  | Concentrated fruit juice |
|  |  | Fruit nectar |
|  |  | Mixed fruit juice |
|  |  | Dehydrated/powdered fruit juice |
|  |  | Vegetable juice |
|  |  | Mixed vegetable juice |
|  |  | Mixed fruit and vegetable juice |
|  | Tea,coffee,  cocoa | Tea (Infusion) |
|  |  | Coffee (beverage) |
|  |  | Coffee imitates beverage |
|  |  | Cocoa beverage |
|  | Drinking water | Drinking water (unspecified) |
|  |  | Tap water |
|  |  | Bottled water |
|  |  | Water ice (for consumption) |
