# Supplementary material for: Dietary changes needed to improve diet sustainability: are they similar across Europe?
Source: Eur J Clin Nutr. 2018 Feb 5;72(7):951–60. doi: 10.1038/s41430-017-0080-z (PMC6035144; doi:10.1038/s41430-017-0080-z)
Supplement: Supplementary file 2 — Contributions of L1 food groups to total diet weight (in grams/day) in observed (OBS), nutritionally adequate diets (NUTR) and nutritionally-adequate diets modeled with a 30% reduction in GHGE(DOCX 48 kb) [file 41430_2017_80_MOESM2_ESM.docx]

**Supplemental Table 2.** Total diet weight and quantities of main food groups and L1 food groups (in grams/day) in observed diets (OBS), nutritionally adequate modeled diets (NUTR) and nutritionally-adequate diets modeled with a 30% reduction in GHGE (NUTR-GHGE-30%).

| **Women** | **France** | | | **UK** | | | **Italy** | | | **Finland** | | | **Sweden** | | |
| --- | --- | --- | --- | --- | --- | --- | --- | --- | --- | --- | --- | --- | --- | --- | --- |
|  | OBS | NUTR | NUTR  GHGE  -30% | OBS | NUTR | NUTR  GHGE  -30% | OBS | NUTR | NUTR  GHGE  -30% | OBS | NUTR | NUTR  GHGE  -30% | OBS | NUTR | NUTR  GHGE  -30% |
| **Total diet weight** | **2464.7** | **2957.6** | **2957.6** | **2444.0** | **2932.8** | **2932.8** | **2061.9** | **2474.3** | **2474.3** | **3066.9** | **3680.2** | **3656.8** | **2563.3** | **2685.9** | **2366.0** |
| **Fruits & vegetables** | **270.9** | **516.2** | **502.7** | **233.8** | **479.1** | **423.8** | **311.0** | **633.2** | **633.2** | **286.0** | **555.2** | **559.4** | **266.5** | **386.4** | **350.3** |
| Fruits | 138.1 | 308.7 | 256.4 | 86.8 | 145.1 | 89.8 | 191.8 | 378.0 | 378.0 | 200.2 | 386.9 | 347.7 | 143.7 | 143.7 | 97.9 |
| Legumes, nuts | 28.4 | 71.4 | 71.4 | 27.3 | 95.3 | 95.3 | 4.5 | 4.5 | 4.5 | 6.7 | 30.8 | 52.9 | 7.0 | 21.7 | 31.4 |
| Vegetables | 104.4 | 136.0 | 174.9 | 119.7 | 238.7 | 238.7 | 114.7 | 250.7 | 250.7 | 79.1 | 137.4 | 158.8 | 115.8 | 221.0 | 221.0 |
| **Starch products** | **238.1** | **236.7** | **288.2** | **256.7** | **273.3** | **308.1** | **176.6** | **219.0** | **205.0** | **258.3** | **439.5** | **439.5** | **236.2** | **301.7** | **318.7** |
| Grains | 190.8 | 164.3 | 189.5 | 177.4 | 272.7 | 272.7 | 152.5 | 194.8 | 180.8 | 232.6 | 413.7 | 413.7 | 200.2 | 265.7 | 282.7 |
| Starchy roots | 47.4 | 72.4 | 98.7 | 79.4 | 0.6 | 35.4 | 24.1 | 24.1 | 24.1 | 25.7 | 25.7 | 25.7 | 36.0 | 36.0 | 36.0 |
| **Plant based**  **mixed dishes** | **128.5** | **197.6** | **85.0** | **65.4** | **188.7** | **188.7** | **345.9** | **156.1** | **319.7** | **226.6** | **103.7** | **262.4** | **149.0** | **45.5** | **138.6** |
| **Dairy & imitates** | **193.6** | **232.2** | **317.4** | **175.6** | **223.1** | **223.3** | **106.8** | **244.1** | **227.3** | **382.4** | **331.1** | **335.0** | **272.4** | **484.3** | **454.7** |
| Cheese | 36.8 | 22.2 | 17.6 | 15.7 | 0.0 | 0.0 | 32.3 | 18.5 | 0.0 | 40.9 | 0.0 | 0.0 | 24.8 | 5.2 | 1.2 |
| Dairy products | 152.5 | 205.7 | 295.5 | 157.3 | 223.1 | 220.7 | 73.5 | 224.6 | 226.3 | 336.8 | 326.4 | 330.3 | 238.5 | 470.0 | 444.4 |
| Dairy imitates | 4.3 | 4.3 | 4.3 | 2.6 | 0.0 | 2.6 | 1.0 | 1.0 | 1.0 | 4.7 | 4.7 | 4.7 | 9.1 | 9.1 | 9.1 |
| **Eggs** | **12.6** | **12.6** | **39.5** | **6.0** | **6.0** | **6.0** | **2.2** | **2.2** | **2.2** | **3.3** | **26.9** | **33.5** | **13.8** | **50.5** | **50.5** |
| **Fish** | **28.0** | **66.6** | **66.6** | **24.8** | **54.8** | **55.2** | **28.6** | **100.0** | **100.0** | **20.0** | **9.2** | **9.2** | **24.1** | **20.9** | **38.2** |
| **Meat & imitates** | **90.9** | **88.6** | **85.4** | **75.4** | **41.3** | **37.5** | **66.7** | **92.5** | **19.5** | **56.9** | **64.2** | **11.7** | **57.9** | **87.8** | **27.8** |
| Livestock meat | 38.1 | 58.3 | 42.1 | 24.3 | 20.1 | 1.7 | 35.9 | 85.8 | 12.9 | 11.7 | 58.8 | 6.3 | 16.8 | 68.4 | 8.4 |
| Meat imitates | 0.1 | 0.1 | 0.1 | 0.7 | 19.3 | 33.9 | 0.1 | 0.1 | 0.1 | 0.3 | 0.3 | 0.3 | 1.2 | 1.2 | 1.2 |
| Other meat | 4.0 | 4.0 | 4.0 | 1.3 | 1.3 | 1.3 | 1.3 | 1.3 | 1.3 | 3.3 | 3.3 | 3.3 | 2.4 | 2.4 | 2.4 |
| Poultry | 22.2 | 22.2 | 22.1 | 27.2 | 0.0 | 0.0 | 13.8 | 0.0 | 0.0 | 16.5 | 0.0 | 0.0 | 15.1 | 0.0 | 0.0 |
| Processed meat | 26.5 | 4.1 | 17.0 | 21.9 | 0.6 | 0.6 | 15.6 | 5.2 | 5.2 | 25.1 | 1.8 | 1.8 | 22.4 | 15.7 | 15.7 |
| **Animal based**  **mixed dishes** | **43.7** | **21.2** | **10.2** | **41.8** | **4.1** | **13.3** | **37.0** | **37.0** | **107.1** | **60.8** | **60.8** | **24.1** | **89.6** | **72.4** | **22.7** |
| **Sugar/fat/alcohol** | **206.0** | **123.6** | **47.7** | **426.1** | **42.8** | **72.6** | **115.5** | **97.2** | **97.2** | **177.9** | **133.9** | **96.4** | **283.4** | **82.7** | **96.0** |
| Animal fats | 9.2 | 0.0 | 0.0 | 3.2 | 0.1 | 0.1 | 0.3 | 0.3 | 0.3 | 1.5 | 0.0 | 0.0 | 0.7 | 0.0 | 0.7 |
| Herbs, spices and condiments | 20.5 | 8.6 | 8.6 | 30.7 | 12.7 | 33.5 | 2.6 | 2.6 | 2.6 | 11.2 | 7.3 | 8.3 | 31.1 | 1.6 | 1.6 |
| Snacks, desserts,  and other foods | 21.2 | 21.2 | 1.0 | 20.9 | 21.3 | 11.3 | 11.8 | 11.8 | 11.8 | 21.0 | 2.3 | 2.3 | 23.8 | 0.0 | 0.0 |
| Sugar and  confectionary | 15.4 | 9.4 | 9.4 | 16.4 | 1.0 | 2.3 | 15.3 | 2.6 | 2.6 | 20.2 | 20.2 | 18.5 | 19.0 | 16.2 | 28.8 |
| Vegetable fats | 12.7 | 7.2 | 8.0 | 8.7 | 7.2 | 7.2 | 5.6 | 0.0 | 0.0 | 21.1 | 1.2 | 0.1 | 9.5 | 0.5 | 0.5 |
| Soft drinks | 70.2 | 20.5 | 0.0 | 208.1 | 0.0 | 0.0 | 21.6 | 21.6 | 21.6 | 39.0 | 39.0 | 39.0 | 103.4 | 0.0 | 0.0 |
| Alcoholic beverages | 56.7 | 56.7 | 20.6 | 138.1 | 0.5 | 18.2 | 58.3 | 58.3 | 58.3 | 63.9 | 63.9 | 28.2 | 95.9 | 64.3 | 64.3 |
| **Water, tea, coffee, juice** | **1252.3** | **1462.2** | **1514.7** | **1138.4** | **1619.4** | **1604.1** | **871.6** | **892.9** | **763.0** | **1594.6** | **1955.8** | **1885.5** | **1170.4** | **1153.8** | **868.4** |
| Drinking water | 794.3 | 954.2 | 1044.1 | 431.6 | 322.7 | 431.6 | 655.3 | 520.6 | 390.7 | 866.4 | 810.5 | 1263.1 | 661.1 | 661.1 | 565.3 |
| Juices | 59.4 | 59.4 | 27.0 | 74.3 | 36.6 | 51.7 | 25.5 | 25.5 | 25.5 | 115.2 | 115.2 | 9.4 | 55.8 | 240.6 | 240.6 |
| Tea,coffee,cocoa | 398.6 | 448.6 | 443.5 | 632.4 | 1260.0 | 1120.8 | 190.8 | 346.8 | 346.8 | 613.0 | 1030.0 | 613.0 | 453.5 | 252.1 | 62.5 |

| **Men** | | France | | | | | | UK | | | | | | Italy | | | | | | Finland | | | | | | Sweden | | | | | |
| --- | --- | --- | --- | --- | --- | --- | --- | --- | --- | --- | --- | --- | --- | --- | --- | --- | --- | --- | --- | --- | --- | --- | --- | --- | --- | --- | --- | --- | --- | --- | --- |
|  | | OBS | | NUTR | | NUTR  GHGE  -30% | | OBS | | NUTR | | NUTR  GHGE  -30% | | OBS | | NUTR | | NUTR  GHGE  -30% | | OBS | | NUTR | | NUTR  GHGE  -30% | | OBS | | NUTR | | NUTR  GHGE  -30% | |
| **Total diet weight** | | **2720.0** | | **3264.0** | | **3205.1** | | **2934.9** | | **3521.9** | | **3521.9** | | **2244.3** | | **2693.1** | | **2637.1** | | **3345.3** | | **3433.2** | | **3166.4** | | **2792.3** | | **3079.4** | | **2718.5** | |
| **Fruits & vegetables** | | **251.2** | | **482.2** | | **467.0** | | **232.4** | | **409.9** | | **355.4** | | **292.3** | | **593.3** | | **508.3** | | **218.9** | | **387.8** | | **407.2** | | **193.9** | | **294.3** | | **328.2** | |
| Fruits | | 124.8 | | 283.3 | | 294.4 | | 86.4 | | 86.4 | | 86.4 | | 169.7 | | 352.0 | | 352.0 | | 145.7 | | 259.8 | | 284.4 | | 97.7 | | 198.1 | | 198.1 | |
| Legumes, nuts | | 32.9 | | 85.7 | | 79.1 | | 31.2 | | 105.0 | | 105.0 | | 4.9 | | 4.9 | | 4.9 | | 5.7 | | 43.4 | | 46.6 | | 5.6 | | 5.6 | | 39.5 | |
| Vegetables | | 93.5 | | 113.2 | | 93.5 | | 114.7 | | 218.5 | | 164.0 | | 117.6 | | 236.3 | | 151.4 | | 67.5 | | 84.6 | | 76.2 | | 90.6 | | 90.6 | | 90.6 | |
| **Starch products** | | **326.7** | | **374.7** | | **368.2** | | **321.6** | | **448.2** | | **522.5** | | **224.4** | | **259.7** | | **295.0** | | **315.1** | | **488.1** | | **387.7** | | **329.0** | | **529.4** | | **487.4** | |
| Grains | | 265.5 | | 246.1 | | 239.6 | | 226.6 | | 334.2 | | 327.0 | | 195.4 | | 230.7 | | 232.2 | | 270.2 | | 295.6 | | 342.8 | | 255.3 | | 276.9 | | 326.4 | |
| Starchy roots | | 61.2 | | 128.6 | | 128.6 | | 95.0 | | 113.9 | | 195.5 | | 29.0 | | 29.0 | | 62.9 | | 44.9 | | 192.5 | | 44.9 | | 73.7 | | 252.5 | | 161.0 | |
| **Plant based**  **mixed dishes** | | **146.8** | | **146.8** | | **59.1** | | **78.5** | | **65.7** | | **48.8** | | **405.9** | | **250.0** | | **302.1** | | **264.7** | | **73.9** | | **73.9** | | **204.6** | | **65.3** | | **47.5** | |
| **Dairy & imitates** | | **194.3** | | **347.1** | | **420.9** | | **200.0** | | **329.9** | | **366.0** | | **88.2** | | **104.5** | | **97.6** | | **448.1** | | **959.4** | | **936.6** | | **321.1** | | **656.0** | | **677.2** | |
| Cheese | | 43.3 | | 0.0 | | 18.5 | | 18.5 | | 0.0 | | 16.2 | | 37.8 | | 54.1 | | 47.2 | | 38.3 | | 27.6 | | 4.8 | | 23.8 | | 2.7 | | 23.8 | |
| Dairy products | | 148.5 | | 344.7 | | 400.0 | | 179.7 | | 328.2 | | 348.0 | | 50.2 | | 50.2 | | 50.2 | | 405.5 | | 927.5 | | 927.5 | | 295.2 | | 651.2 | | 651.2 | |
| Dairy imitates | | 2.4 | | 2.4 | | 2.4 | | 1.7 | | 1.7 | | 1.7 | | 0.1 | | 0.1 | | 0.1 | | 4.3 | | 4.3 | | 4.3 | | 2.1 | | 2.1 | | 2.1 | |
| **Eggs** | | **12.6** | | **12.6** | | **43.5** | | **6.6** | | **6.6** | | **6.6** | | **2.5** | | **2.5** | | **2.5** | | **6.6** | | **6.6** | | **6.6** | | **13.3** | | **31.7** | | **56.0** | |
| **Fish** | | **28.2** | | **64.4** | | **72.1** | | **27.0** | | **30.1** | | **20.4** | | **28.5** | | **70.3** | | **74.2** | | **26.1** | | **12.4** | | **12.4** | | **25.0** | | **22.8** | | **13.1** | |
| **Meat & imitates** | | **138.1** | | **158.1** | | **169.9** | | **108.6** | | **96.4** | | **67.8** | | **87.4** | | **75.6** | | **47.0** | | **105.0** | | **28.0** | | **25.4** | | **92.7** | | **105.5** | | **44.2** | |
| Livestock meat | | 59.8 | | 110.5 | | 59.6 | | 34.9 | | 92.7 | | 25.9 | | 46.1 | | 46.1 | | 14.9 | | 19.8 | | 19.8 | | 17.2 | | 29.5 | | 77.8 | | 35.9 | |
| Meat imitates | | 0.1 | | 0.1 | | 0.1 | | 1.0 | | 1.0 | | 39.2 | | 0.0 | | 0.0 | | 0.0 | | 0.1 | | 0.1 | | 0.1 | | 0.7 | | 0.7 | | 0.7 | |
| Other meat | | 5.3 | | 5.3 | | 5.3 | | 1.9 | | 1.9 | | 1.9 | | 1.3 | | 1.3 | | 1.3 | | 6.7 | | 6.7 | | 6.7 | | 5.0 | | 5.0 | | 5.0 | |
| Poultry | | 33.8 | | 33.8 | | 96.4 | | 33.1 | | 0.0 | | 0.0 | | 17.2 | | 17.2 | | 17.2 | | 23.5 | | 0.0 | | 0.0 | | 19.7 | | 19.7 | | 0.0 | |
| Processed meat | | 39.2 | | 8.5 | | 8.5 | | 37.7 | | 0.8 | | 0.8 | | 22.8 | | 11.0 | | 13.6 | | 54.8 | | 1.3 | | 1.3 | | 37.9 | | 2.4 | | 2.7 | |
| **Animal based**  **mixed dishes** | | **64.4** | | **11.6** | | **11.6** | | **69.7** | | **15.8** | | **69.2** | | **53.0** | | **53.0** | | **53.0** | | **89.3** | | **16.6** | | **16.6** | | **124.2** | | **6.2** | | **6.2** | |
| **Sugar and fats** | | **397.9** | | **380.1** | | **285.6** | | **777.3** | | **366.0** | | **253.2** | | **235.2** | | **234.1** | | **132.3** | | **352.4** | | **314.3** | | **326.9** | | **470.2** | | **440.3** | | **405.1** | |
| Animal fats | | 10.5 | | 0.0 | | 0.0 | | 3.3 | | 0.1 | | 0.1 | | 0.4 | | 0.4 | | 0.4 | | 3.6 | | 0.0 | | 0.0 | | 0.8 | | 0.8 | | 0.8 | |
| Herbs, spices and condiments | | 21.1 | | 14.3 | | 10.4 | | 33.2 | | 33.2 | | 10.0 | | 2.3 | | 2.3 | | 2.3 | | 18.7 | | 4.5 | | 4.5 | | 36.7 | | 6.8 | | 6.8 | |
| Snacks, desserts, and other foods | | 23.1 | | 23.1 | | 1.3 | | 24.8 | | 37.3 | | 14.7 | | 13.9 | | 13.9 | | 13.9 | | 17.4 | | 17.4 | | 17.4 | | 25.1 | | 25.1 | | 25.1 | |
|  | |  | |  | |  | |  | |  | |  | |  | |  | |  | |  | |  | |  | |  | |  | |  | |
| Sugar and confectionary | | 19.0 | | 19.0 | | 19.0 | | 24.5 | | 2.6 | | 2.6 | | 17.6 | | 17.6 | | 17.6 | | 21.4 | | 21.4 | | 21.4 | | 15.0 | | 15.0 | | 15.0 | |
| Vegetable fats | | 12.1 | | 11.6 | | 13.6 | | 11.0 | | 25.5 | | 25.5 | | 5.5 | | 4.5 | | 5.5 | | 26.2 | | 5.9 | | 18.5 | | 12.2 | | 12.2 | | 3.6 | |
| Soft drinks | | 111.0 | | 111.0 | | 111.0 | | 264.4 | | 231.1 | | 188.1 | | 32.0 | | 32.0 | | 32.0 | | 77.7 | | 77.7 | | 77.7 | | 154.3 | | 154.3 | | 227.5 | |
| Alcoholic beverages | | 201.1 | | 201.1 | | 130.3 | | 416.1 | | 36.2 | | 12.1 | | 163.5 | | 163.5 | | 60.6 | | 187.4 | | 187.4 | | 187.4 | | 225.9 | | 225.9 | | 126.1 | |
| **Water, tea, coffee, juice** | | **1159.8** | | **1286.4** | | **1307.2** | | **1113.2** | | **1753.1** | | **1812.0** | | **826.9** | | **1050.1** | | **1125.2** | | **1519.0** | | **1146.0** | | **973.0** | | **1018.2** | | **927.9** | | **653.6** | |
| Drinking water | | 765.8 | | 765.8 | | 796.7 | | 404.9 | | 445.8 | | 1059.1 | | 616.0 | | 682.4 | | 757.5 | | 656.4 | | 286.7 | | 586.7 | | 495.5 | | 405.1 | | 495.5 | |
| Juices | | 58.4 | | 70.7 | | 91.1 | | 78.7 | | 79.8 | | 78.7 | | 27.7 | | 27.7 | | 27.7 | | 166.3 | | 163.0 | | 166.3 | | 69.1 | | 69.1 | | 69.1 | |
| Tea,coffee,cocoa | | 335.5 | | 449.8 | | 419.3 | | 629.5 | | 1227.5 | | 674.1 | | 183.2 | | 340.0 | | 340.0 | | 696.3 | | 696.3 | | 220.0 | | 453.6 | | 453.6 | | 89.0 | |
